# Pre-injury antithrombotic agents predict intracranial hemorrhagic progression, but not worse clinical outcome in severe traumatic brain injury

**DOI:** 10.1007/s00701-021-04816-0

**Published:** 2021-03-26

**Authors:** Teodor Svedung Wettervik, Samuel Lenell, Per Enblad, Anders Lewén

**Affiliations:** Department of Neuroscience, Section of Neurosurgery, Uppsala University, SE-751 85 Uppsala, Sweden

**Keywords:** Anticoagulants, Antiplatelet, Antithrombotics, Clinical outcome, Hemorrhagic progression, Traumatic brain injury

## Abstract

**Background:**

The incidence of traumatic brain injury (TBI) patients of older age with comorbidities, who are pre-injury treated with antithrombotic agents (antiplatelets and/or anticoagulants), has increased. In this study, our aim was to investigate if pre-injury antithrombotic treatment was associated with worse intracranial hemorrhagic/injury progression and clinical outcome in patients with severe TBI.

**Methods:**

In this retrospective study, including 844 TBI patients treated at our neurointensive care at Uppsala University Hospital, Sweden, 2008–2018, 159 (19%) were pre-injury treated with antithrombotic agents. Demography, admission status, radiology, treatment, and outcome variables were evaluated. Significant intracranial hemorrhagic/injury evolution was defined as hemorrhagic progression seen on the second computed tomography (CT), emergency neurosurgery after the initial CT, or death following the initial CT.

**Results:**

Patients with pre-injury antithrombotics were significantly older and with a higher Charlson comorbidity index. They were more often injured by falls and more frequently developed acute subdural hematomas. Sixty-eight (8%) patients were pre-injury treated with monotherapy of antiplatelets, 67 (8%) patients with anticoagulants, and 24 (3%) patients with a combination of antithrombotics. Pre-injury anticoagulants, but not antiplatelets, were independently associated with significant intracranial hemorrhagic/injury evolution in a multiple regression analysis. However, neither anticoagulants nor antiplatelets were associated with mortality and unfavorable outcome in multiple regression analyses.

**Conclusions:**

Only anticoagulants were associated with intracranial hemorrhagic/injury progression, but no antithrombotic agent correlated with worse clinical outcome. Management, including early anticoagulant reversal, availability of emergency neurosurgery, and neurointensive care, may be important aspects for reducing the adverse effects of pre-injury antithrombotics.

**Supplementary Information:**

The online version contains supplementary material available at 10.1007/s00701-021-04816-0.

## Introduction

The epidemiology of traumatic brain injury (TBI) is changing. As the population ages, the incidence of old TBI victims with pre-injury comorbidities has increased [18, 19, 31, 32]. This implicates new challenges in TBI care, and one particular concern is the pre-injury use of antithrombotic agents. Coagulopathy is a feared complication to TBI and may be further aggravated by pre-injury antithrombotic treatment [20]. However, there is controversy if the negative effect of antiplatelets on platelet function is sufficient to cause increased hemorrhagic progression and to affect clinical outcome after head trauma [4, 7, 10, 13, 14, 16, 22, 26, 28, 33, 36–38]. The current guidelines recommend antiplatelet withdrawal in case of post-traumatic intracranial hemorrhage, but there is limited knowledge if and when more active reversal agents, including platelet transfusion or desmopressin, are indicated [2, 12]. Most studies on pre-injury vitamin K antagonists treatment demonstrate an increased risk of hemorrhagic progression of intracranial lesions and increased mortality following head trauma [3, 4, 6, 11, 14, 26, 28, 29, 36], but early vitamin K antagonists reversal may efficiently counteract these adverse effects [12, 15]. Some recent studies even suggest that pre-injury vitamin K antagonists no longer impact outcome to a significant extent due to modern management [18, 22]. Novel oral anticoagulants (NOAC) has increased in incidence and replaced vitamin K antagonists to some degree [1]. There are concerns that they would favor worse hemorrhagic progression of intracranial lesions after head trauma due to a lack of effective antidotes for most NOACs [12]. However, recent studies demonstrate a lower risk of hemorrhagic progression and better clinical outcome in comparison to patients with pre-injury vitamin K antagonists in TBI [28, 29]. Furthermore, a subgroup of patients with a severe risk of thromboembolic complications are treated with a combination of antithrombotic agents, such as dual antiplatelets or an antiplatelet together with an anticoagulant. These patients may have an even higher risk of hemorrhagic progression of intracranial lesions and worse clinical outcome [28].

Hence, there are still questions regarding the effect of different pre-injury antithrombotic agents on intracranial hemorrhage progression and clinical outcome after head trauma. Most previous findings are based on patient cohorts from the emergency department including mild TBI cases who did not require neurosurgical interventions. In the current study, we therefore aimed to evaluate the incidence of antithrombotic agents in an updated patient population with severe TBI treated at our tertiary neurointensive care unit, their effect on hemorrhagic progression of intracranial lesions, and the relation to clinical outcome. Our hypotheses were that antithrombotic agents were relatively common, associated with severe hemorrhagic progression, and worse clinical outcome.

## Materials and methods

### Patients

The Department of Neurosurgery at the University Hospital in Uppsala provides neurosurgical care for a central part of Sweden, with a catchment population around 2 million people. Most patients are resuscitated at their local hospitals and then referred to our neurointensive care unit (the most distant local hospital 382 km away). There were 926 TBI patients aged 15 or older who were treated at our neurointensive care unit between 2008 and 2018 eligible for inclusion in this retrospective study. Seventy-five patients were excluded since they had been treated at another neurointensive care unit before admission to our department if they were discharged to a different catchment area/country with another neurosurgery department. Seven patients were treated twice at our neurointensive care and were therefore registered twice, but we only included data from their first neurointensive care visit. Hence, the final study population included 844 TBI patients.

### Management protocol

Patients were treated in accordance with our standardized intracranial pressure (ICP)- and cerebral perfusion pressure-oriented treatment protocol to avoid secondary insults, as previously described in detail [9, 39]. Unconscious (GCS M < 6) patients were intubated, mechanically ventilated, and received ICP monitoring. Treatment goals were ICP ≤ 20 mm Hg, cerebral perfusion pressure ≥ 60 mm Hg, systolic blood pressure > 100 mm Hg, central venous pressure 0–5 mm Hg, pO_2_ > 12 kPa, arterial glucose 5–10 mmol/L (mM), hemoglobin > 100 g/L, electrolytes within normal ranges, normovolemia, and body temperature < 38°C. Patients were initially mildly hyperventilated (4.0–4.5 kPa) and normoventilated as soon as ICP allowed.

For patients with pre-injury antithrombotic agent treatment, the antithrombotic agent was withdrawn following TBI. Antiplatelets were generally only withdrawn but was occasionally reversed with thrombocytes and/or desmopressin. Vitamin K antagonists were reversed with vitamin K and prothrombin complex concentrate. NOAC was withdrawn and sometimes treated with cyklokapron and/or prothrombin complex concentrate.

### Data acquisition and analysis

Demographic, admission, and treatment variables were collected from the Uppsala TBI register [24]. The extent of comorbidities was evaluated according to the Charlson comorbidity index [34]. Routine blood and coagulation status including hemoglobin, platelets, PK-INR, and APTT at admission were evaluated. The testing was done at the accredited laboratory of the Department of Clinical Chemistry at Uppsala University Hospital.

Computed tomography (CT) scans of the brain were evaluated according to the Marshall classification [21]. The size of intracranial hemorrhages was evaluated and compared on the first two CT scans. Hemorrhage progression of intracranial lesions was defined similarly to Shin et al. [29], as an increase on the second CT in (1) epidural hematoma (EDH) or acute subdural hematoma (ASDH) width with more than 2 mm, (2) traumatic subarachnoid hemorrhage (tSAH) by visual inspection, (3) intraventricular hemorrhage (IVH) with more than 2 mm in lateral width, and/or (4) cerebral contusions with more than 6 mL or 33%. The contusion volume was calculated according to the ABC/2 formula [17]. However, some patients received emergency neurosurgery immediately after the first CT, and some patients had large intracranial hemorrhages and were in such a poor clinical condition that they were not considered to benefit from surgery and developed total brain death before a second CT was done. To take into account these different clinical trajectories of hemorrhage/injury evolution, we divided the patients into four groups: (1) stable intracranial hemorrhages on follow-up CT, (2) progression of intracranial hemorrhages as defined above on follow-up CT, (3) immediate intracranial surgery after the first CT, and (4) total brain infarction (brain death) confirmed before a second CT. For statistical purposes, we also dichotomized these groups into stable intracranial lesions at follow-up CT (group 1) and significant intracranial hemorrhage/injury evolution (groups 2, 3, and 4).

Clinical outcome was assessed by specially trained personnel with structured telephone interviews at 6 months post-injury using the Extended Glasgow Outcome Scale (GOS-E), containing eight categories of global outcome, from death to upper good recovery [24, 35, 40]. The interviews were held with the patients if they had recovered sufficiently, otherwise, with their next of kin.

### Statistical analysis

Demography, admission status, coagulation status, treatments, and clinical outcome were described as median (interquartile range) or number (proportion). The Mann-Whitney *U*-test and Pearson’s chi-square analysis were used for statistical comparisons between patients that were pre-injury treated with ATs and those who did not receive such treatment. Missing values were rare, and those were excluded from the analyses.

Differences in hemorrhagic/injury progression of intracranial lesions (stable, progression, emergency surgery, or total brain infarction) in relation to pre-injury antithrombotic agents were evaluated with Pearson’s Chi-square analysis. A similar sub-analysis was done for different types of antithrombotic agents (antiplatelets, anticoagulants, and multiple/a combination of antithrombotic agents). Multiple logistic regression analyses were done to evaluate the risk of significant intracranial hemorrhage/injury progression (groups 2, 3, and 4, i.e., progression of lesions on follow-up CT, immediate neurosurgery, or total brain infarction) using only pre-injury variables age, Charlson comorbidity index, and antithrombotic agents in combination with mechanism of injury as explanatory variables. A similar multiple logistic regression analysis was done with the various antithrombotic subtypes instead of antithrombotic agents (as one group) as explanatory variables for significant intracranial hemorrhage progression. In addition to hemorrhage evolution, brain edema is another contributing cause for emergency neurosurgery. We therefore evaluated if exclusion of those patients with the supposedly worst early brain edema development (defined as those operated with primary DC and those with immediate brain death after the first CT) had any impact upon the results.

The relation between antithrombotic agents and mortality and favorable clinical outcome at 6 months was evaluated with Pearson’s chi-square test. Multiple logistic regression analyses were also performed for mortality and favorable outcome, respectively, as dependent variables and pre-injury variables (age, Charlson comorbidity index, and antithrombotic agents) in combination with mechanism of injury as explanatory variables. A similar multiple logistic regression analysis was done using the antithrombotic subtypes instead of antithrombotic agents (as one group) as explanatory variables for mortality and favorable clinical outcome at 6 months. Similar regression analyses of clinical outcome with the traditional IMPACT core variables [30] as explanatory variables were also performed. A *p*-value < 0.05 was considered statistically significant.

## Results

### Demography, admission status, and treatments

Descriptive clinical data for the entire TBI patient population is described in Table 1. There were 844 TBI patients included in the study, of which 159 (19%) patients were pre-injury treated with antithrombotic agents, whereas 685 (81%) patients were not on such agents. The differences in demography, admission status, and treatments between the two groups are also described in Table 1.

**Table 1:** Demographic, admission, treatments, and outcome in relation to pre-injury treatment with antithrombotics

	All	Antithrombotics	No antithrombotics	*p*-value
Patients, *n* (%)	844	159 (19%)	685 (81%)	NA
Age, median (IQR)	54 (34–67)	72 (65–77)	49 (28–62)	0.001
Sex (female/male)	198/646 (24/76)	37/122 (23/73%)	161/524 (24/76%)	1.00
Charlson comorbidity index, median (IQR)	0 (0–0)	1 (0–3)	0 (0–0)	0.001
Injury mechanism				0.001
Fall, *n* (%)	494 (59%)	136 (86%)	358 (52%)	
Vehicle accident, *n* (%)	172 (20%)	9 (6%)	163 (24%)	
Bicycle accident, *n* (%)	65 (8%)	6 (4%)	59 (9%)	
Pedestrian, *n* (%)	29 (3%)	3 (2%)	26 (4%)	
Assault, *n* (%)	36 (4%)	2 (1%)	34 (5%)	
Sport accident, *n* (%)	14 (2%)	1 (1%)	13 (2%)	
Other, *n* (%)	34 (4%)	2 (1%)	32 (5%)	
Intracranial hemorrhage				
EDH, *n* (%)	88 (11%)	3 (2%)	85 (13%)	0.001
ASDH, *n* (%)	376 (46%)	119 (76%)	257 (38%)	0.001
tSAH, *n* (%)	522 (63%)	78 (50%)	444 (66%)	0.001
IVH, *n* (%)	91 (11%)	16 (10%)	75 (11%)	0.89
Contusion, *n* (%)	180 (22%)	34 (22%)	146 (22%)	1.00
Intracranial hemorrhage/injury evolution (yes/no), *n* (%)	409/435 (48/52%)	103/56 (65/35%)	306/379 (45/55%)	0.001
Marshall grade				0.001
Diffuse injury I, *n* (%)	7 (1%)	0 (0%)	7 (1%)	
Diffuse injury II, *n* (%)	477 (56%)	58 (36%)	419 (61%)	
Diffuse injury III, *n* (%)	35 (4%)	0 (0%)	35 (5%)	
Diffuse injury IV, *n* (%)	33 (4%)	12 (8%)	21 (3%)	
Evacuated mass lesion, *n* (%)	205 (24%)	56 (35%)	149 (22%)	
Non-evacuated mass lesion, *n* (%)	87 (10%)	33 (21%)	54 (8%)	
GCS M, median (IQR)	5 (5-6)	6 (5-6)	5 (5-6)	0.001
Pupillary abnormalities, *n* (%)	128 (15%)	25/134 (16/84%)	103/82 (15/85%)	0.81
Hemoglobin (g/L), median (IQR)	126 (113-139)	124 (110-134)	127 (113-139)	0.06
Platelets (10^9^/L), median (IQR)	216 (162-273)	213 (160-271)	216 (163-274)	0.99
PK-INR, median (IQR)	1.1 (1.0-1.2)	1.2 (1.0-1.4)	1.1 (1.0-1.2)	0.001
APTT (s), median (IQR)	33 (31-37)	36 (32-42)	33 (31-36)	0.001
ICP monitor (none/Codman/EVD/both), *n* (%)	327/304/102/111 (39/36/12/13%)	82/53/13/11 (52/33/8/7%)	245/251/89/100 (36/37/13/15%)	0.001
Craniotomies, median (IQR)	0 (0-1)	1 (0-1)	0 (0-1)	0.001
Decompressive craniectomy, *n* (%)	57 (7%)	2/157 (1/99%)	55/630 (8/92%)	0.001
Thiopental, *n* (%)	64 (8%)	2/157 (1/99%)	62/623 (9/91%)	0.001
Mortality at 6 months, *n* (%)	134 (17%)	55 (37%)	79 (13%)	0.001
Favorable/unfavorable clinical outcome at 6 months, *n* (%)	464/315 (40/60%)	60/89 (40/60%)	404/226 (64/36%)	0.001

*ASDH* acute subdural hematoma, *EDH* epidural hematoma, *GCS M* Glasgow Coma Scale Motor Score, *ICP* intracranial pressure, *IQR* interquartile range, *IVH* intraventricular hemorrhage, *tSAH* traumatic subarachnoid hemorrhage

### Antithrombotics—types of agents, indication, coagulation status, and reversal treatment

**Fig. 1:**
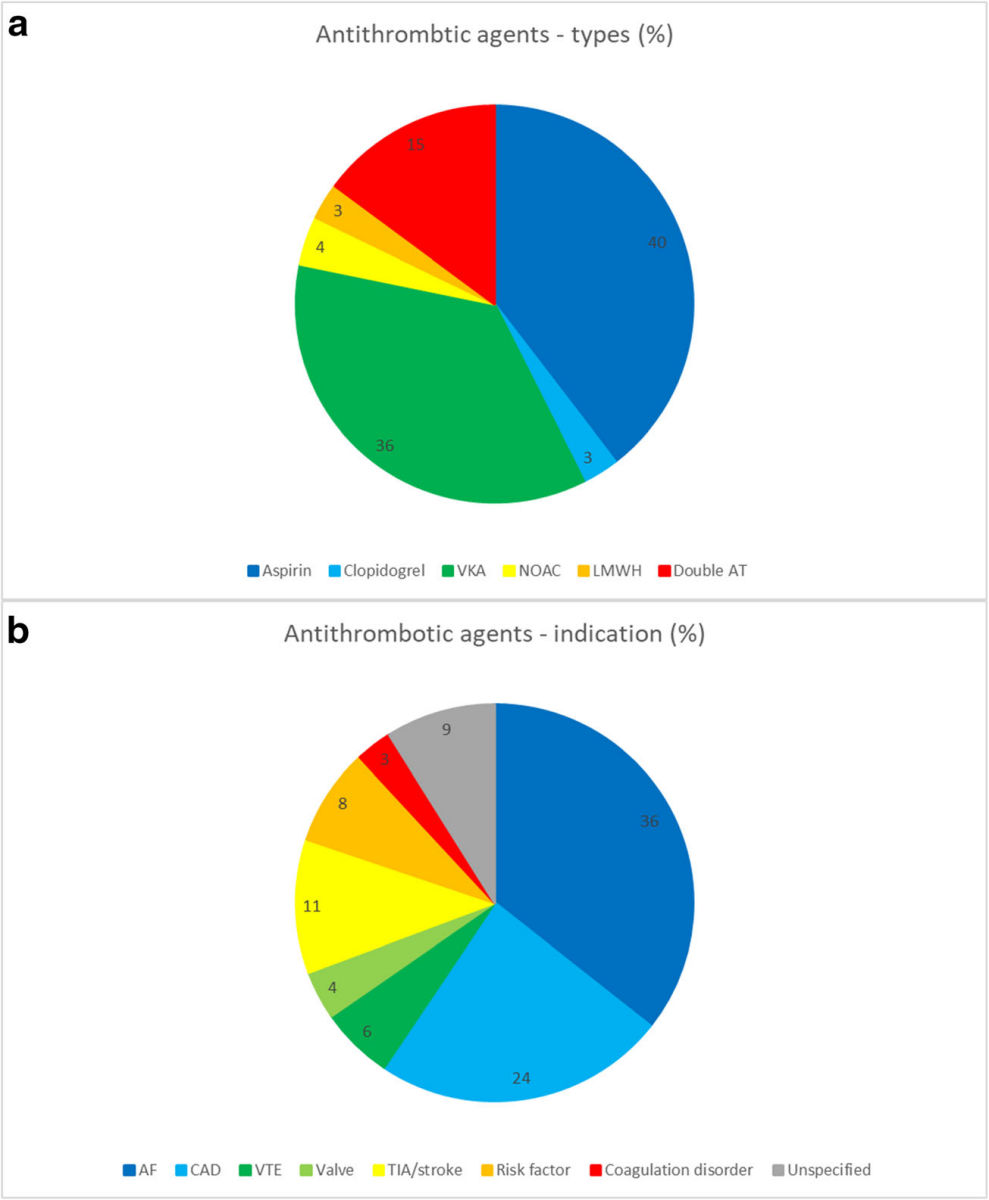
Types of antithrombotic agents and indications for treatment. AF, atrial fibrillation; AT, antithrombotic; CAD, coronary artery disease; LMWH, low-molecular weight heparin; NOAC, novel oral anticoagulant; VKA, vitamin K antagonist; VTE, venous thromboembolism

The various types of pre-injury antithrombotic agents and their indications are described in Fig. 1. Eighty-nine (56%) of the patients with antithrombotic agents were pre-injury treated with antiplatelets. Sixty-three (40%) patients had aspirin as monotherapy, 5 (3%) patients had clopidogrel as monotherapy, and 21 (13%) patients had an antithrombotic combination of an antiplatelet together with another antithrombotic agent (antiplatelet or anticoagulant). Eighty-two (52%) of the 159 patients with antithrombotic agents were pre-injury treated with anticoagulants. Fifty-seven (36%) patients had a vitamin K antagonist as monotherapy, 6 (4%) patients had NOAC as monotherapy, 5 (3%) patients had low-molecular weight heparin as monotherapy, and 15 (9%) had an antithrombotic combination of an anticoagulant together with another antithrombotic agent (antiplatelet or anticoagulant). There were in total 24 (15%) patients with a combination of pre-injury antithrombotic agent. Ten (6%) patients received dual antiplatelets (aspirin + P2Y_12_-inhibitor), 12 (8%) patients received a combination of antiplatelet and anticoagulant (9 patients with aspirin + vitamin K antagonist, 2 patients with aspirin + low-molecular weight heparin, and 1 patient with clopidogrel + heparin), and 2 (1%) patients received dual anticoagulants (1 patient with vitamin K antagonist + low-molecular weight heparin and 1 patient with low-molecular weight heparin + fondaparinux/Arixtra). The reversal management of these different antithrombotic agents is described in Table 2.

**Table 2:** Antithrombotic agents and reversal management

Antithrombotic regime	Antithrombotic agent	Patients, *n*	Reversal	Patients, *n* (%)
Antiplatelet	Aspirin	63	Withdrawal	58 (92%)
			Platelet transfusion and desmopressin	4 (6%)
			Protein complex concentration	1 (2%)
	Clopidogrel	5	Withdrawal	4 (80%)
			Platelet transfusion and desmopressin	1 (20%)
Anticoagulants	Vitamin K antagonist	57	Withdrawal	1 (2%)
			Vitamin K + protein complex concentration	56 (98%)
	NOAC	6	Withdrawal	1 (17%)
			Praxbind	1 (17%)
			Tranexamic acid + protein complex concentration	1 (17%)
			Vitamin K + protein complex concentration	3 (50%)
	LMWH	5	Withdrawal	4 (80%)
			Plasma transfusion	1 (20%)
Antithrombotic combination	Dual antiplatelets	10	Withdrawal	5 (50%)
			Platelet transfusion and desmopressin	5 (50%)
	Aspirin + vitamin K antagonist	9	Vitamin K + protein complex concentration	9 (100%)
	Aspirin + LMWH	2	Withdrawal	2 (100%)
	Clopidogrel + heparin	1	Withdrawal	1 (100%)
	Dual anticoagulants	2	Vitamin K + protein complex concentration/plasma	2 (100%)

*LMWH* low-molecular weight heparin, *NOAC* novel oral anticoagulant

Those with pre-injury antithrombotic agents had significantly higher PK-INR (median 1.2 IQR 1.0–1.4 vs. 1.1 IQR 1.0–1.2, *p*-value 0.001) and higher APTT (median 36 IQR 32–41 vs. 33 IQR 31–36, *p*-value = 0.001) at neurointensive care admission. There was no difference in hemoglobin (median 124 IQR 110–134 vs. 127 IQR 113–139, *p*-value = 0.06) or platelets (median 213 IQR 160–271 vs. 216 IQR 163–274, *p*-value = 0.99).

### Antithrombotic agents—relation to post-traumatic intracranial hemorrhage/injury evolution

**Fig. 2:**
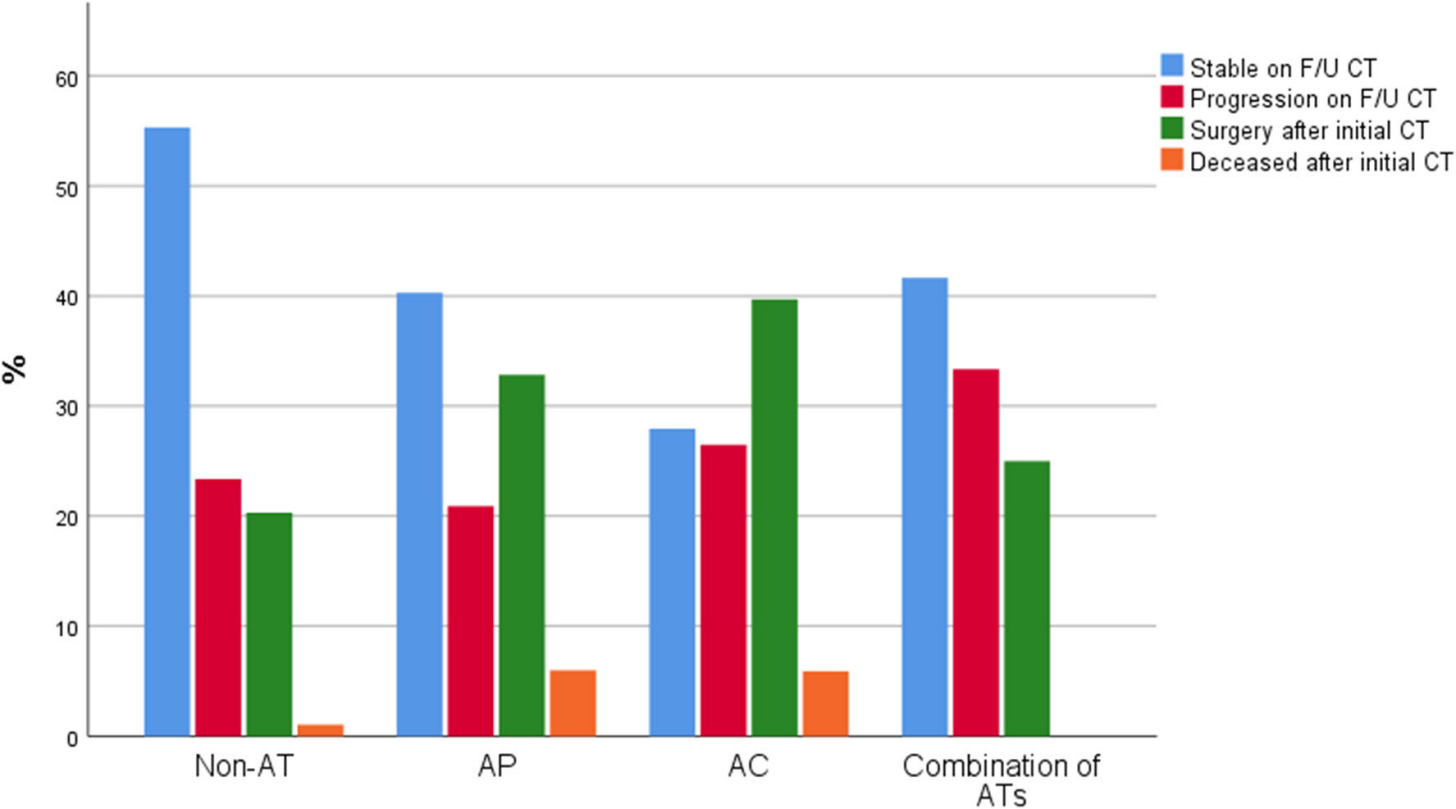
Antithrombotic agents in relation to hemorrhagic progression, the need for immediate surgery, and death. The figure demonstrates the relation among antithrombotic agents and intracranial hemorrhage progression, defined as either stable on F/U CT (a.k.a. group 1), significant progression on F/U CT (group 2), emergency neurosurgery after initial CT (group 3), and deceased after the initial CT (group 4). In the non-AT group, the number of patients in group 1/2/3/4 was 379/160/139/7 patients. In the AP group, the number of patients in group 1/2/3/4 was 27/14/22/4. In the AC group, the number of patients in group 1/2/3/4 was 19/18/27/4. In the combination of ATs group, the number of patients in group 1/2/3/4 was 10/8/6/0. Eighty-six (43%) of 200 patients with significant hemorrhage progression on F/U CT (group 2) required a craniotomy for hematoma evacuation later. AC, anticoagulant; AP, antiplatelet; AT, antithrombotic agent; F/U, follow-up; CT, computed tomography

Patients with pre-injury antithrombotic agents were significantly less likely to have a hemorrhage that was stable at follow-up CT than those without pre-injury antithrombotic treatment (35%, vs. 55%, *p*-value = 0.001). In a sub-analysis, the risk of having a significant post-traumatic intracranial hemorrhage/injury evolution was independently associated with pre-injury treatment with anticoagulants (Fig. 2 and Table 3). Antiplatelets or a combination of two antithrombotic agents were not independent risk factors for significant post-traumatic intracranial hemorrhage evolution. Lower age and a vehicle accident rather than falls as the mechanism of injury were independently associated with a lower risk of significant post-traumatic intracranial hemorrhage/injury evolution. Similar associations in the regressions were found if those with primary DC (*n* =29) and those who developed early brain death after the first CT (*n* = 15) were excluded from the analyses. The second CT was in median performed 10 h (IQR 6–20) after the first CT.

**Table 3:** Antithrombotic agents and the risk of significant intracranial hemorrhage/injury evolution (progression, immediate surgery, and total brain infarction)—a multiple logistic regression analysis

Variables	Regression 1—significant hemorrhage		Regression 2—significant hemorrhage	
	OR (95% CI)	*p*-value	OR (95% CI)	*p*-value
Age	**1.02 (1.01–1.03)**	*0.001*	**1.02 (1.01–1.03)**	*0.001*
Charlson comorbidity index	0.99 (0.86–1.13)	0.84	1.00 (0.87–1.15)	0.97
Antithrombotic agent (yes)	1.27 (0.81–1.99)	0.29	NA	NA
Antiplatelet (yes)	NA	NA	0.98 (0.56–1.73)	0.94
Anticoagulant (yes)	NA	NA	**1.94 (1.08–3.47)**	*0.03*
Combination of antithrombotics (yes)	NA	NA	0.61 (0.23–1.65)	0.33
Injury mechanism (fall—reference)		*0.01*		*0.004*
Vehicle accident	**0.43 (0.29–0.65)**	*0.001*	**0.42 (0.28–0.63)**	*0.001*
Bicycle accident	0.64 (0.38–1.10)	0.11	0.65 (0.38–1.11)	0.11
Pedestrian	0.78 (0.36–1.68)	0.78	0.77 (0.36–1.67)	0.51
Assault	0.57 (0.27–1.20)	0.14	0.55 (0.26–1.15)	0.11
Sport accident	0.85 (0.28–2.57)	0.77	0.82 (0.27–2.49)	0.72
Other	0.96 (0.47–1.95)	0.90	0.94 (0.46–1.92)	0.07

The multiple logistic regression analyses describe the explanatory variables for significant intracranial hemorrhage/injury progression (defined as significant hemorrhage progression on follow-up CT, immediate hematoma evacuation after the first CT, or immediate death after the first CT) in contrast to stable intracranial hemorrhage on follow-up CT. Pre-injury treatment with antithrombotic agents was grouped as one entity in regression 1, whereas different antithrombotic subtypes (antiplatelets, anticoagulants, and having a combination of antithrombotics) were analyzed in regression 2. *CI* confidence interval, *CT* computed tomography, *NA* not applicable

Bold and italics indicate statistical significance

### Antithrombotics—relation to mortality and favorable clinical outcome

Pre-injury antithrombotic agents were significantly associated with increased mortality (37% vs. 13%, *p*-value = 0.001) and decreased rate of favorable clinical outcome (40% vs. 64%, *p*-value = 0.001) at 6 months in univariate analyses (Table 1). However, in a multiple logistic regression analysis, only higher age and higher Charlson comorbidity index were associated with higher mortality and decreased rate of favorable clinical outcome, whereas pre-injury antithrombotic agents were not (Table 4). Similarly, a sub-analysis of antiplatelets, anticoagulants, and a combination of antithrombotic agents in the same regression instead of antithrombotics (as one group) did not reveal any significant association with mortality or favorable clinical outcome. Similarly, pre-injury antithrombotic agents were not independently associated with mortality or favorable clinical outcome in regressions including the IMPACT core variables (Supplementary, Appendix A).

**Table 4:** Antithrombotic agents in relation to mortality and favorable clinical outcome—a multiple logistic regression analysis

Variables	Regression 1—mortality		Regression 2—mortality	
	OR (95% CI)	*p*-value	OR (95% CI)	*p*-value
Age	**1.04 (1.03–1.06)**	*0.001*	**1.04 (1.03–1.06)**	*0.001*
Charlson comorbidity index	**1.39 (1.19–1.63)**	*0.001*	**1.42 (1.21–1.67)**	*0.001*
Antithrombotic agent (yes)	1.20 (0.72–2.02)	0.49	NA	NA
Antiplatelet (yes)	NA	NA	0.87 (0.45–1.71)	0.69
Anticoagulant (yes)	NA	NA	1.37 (0.75–2.53)	0.31
Combination of antithrombotics (yes)	NA	NA	0.88 (0.29–2.66)	0.83
Mechanism of injury (7 categories)	NA	0.69	NA	0.70
**Variables**	**Regression 1—favorable outcome**		**Regression 2—favorable outcome**	
	**OR (95% CI)**	*p*-value	**OR (95% CI)**	*p*-value
Age	**0.97 (0.96–0.98)**	*0.001*	**0.97 (0.96–0.98)**	*0.001*
Charlson comorbidity index	**0.84 (0.72–0.98)**	*0.02*	**0.81 (0.69–0.95)**	*0.01*
Antithrombotic agent (yes)	1.01 (0.64–1.60)	0.96	NA	NA
Antiplatelet (yes)	NA	NA	1.36 (0.75–2.46)	0.31
Anticoagulant (yes)	NA	NA	0.91 (0.51–1.62)	0.74
Combination of antithrombotics (yes)	NA	NA	1.42 (0.51–3.95)	0.50
Mechanism of injury (7 categories)	NA	0.44	NA	0.45

The multiple logistic regression analyses describe the explanatory variables for mortality and favorable outcome, respectively. Pre-injury treatment with antithrombotic agents was grouped as one entity in regression 1, whereas different antithrombotic subtypes (antiplatelets, anticoagulants, and having a combination of antithrombotics) were analyzed in regression 2. *CI* confidence interval, *NA* not applicable

Bold and italics indicate statistical significance

## Discussion

In the current study including 844 patients with severe TBI treated at our neurointensive care unit between 2008 and 2018, we found that those with pre-injury antithrombotic agents, particularly anticoagulants but not pre-injury antiplatelets, were more likely to suffer from significant post-traumatic intracranial hemorrhage evolution. The rate of anticoagulant reversal was high, and the patients often required emergency neurosurgery. Interestingly, neither anticoagulants nor antiplatelets were independently associated with increased mortality or decreased rate of favorable clinical outcome. The main conclusion is that caution is needed due to the risk of significant hemorrhagic progression of intracranial lesions in patients with pre-injury anticoagulants, but favorable outcome may be achieved. Early anticoagulant reversal, emergency neurosurgery, and neurointensive care may be important aspects to counteract the adverse effects of antithrombotic agents in case of TBI.

### Antithrombotics and post-traumatic intracranial hemorrhagic progression

Traumatic forces to the head pose a risk of neurovascular disruption with development of intracranial hemorrhages. This risk may increase due to trauma-induced coagulopathy and pre-injury treatment with antithrombotic agents [20]. As the general population is aging, the incidence of TBI patients who are older has comorbidities, and pre-injury antithrombotic agents is also rising [1, 18, 19]. There is hence a need for a better understanding of post-traumatic intracranial hemorrhage evolution for patients treated with pre-injury antithrombotic agents and for specific antithrombotic types, to better appreciate the risk for severe deterioration and clinical outcome.

Although antithrombotic agents may induce a worsening of primary hemostasis and/or coagulation, the risk of severe antithrombotic agent-induced coagulopathy and significant hemorrhage progression in general [5, 25] and following head trauma specifically [7, 10, 16, 22, 26, 28, 38] remain controversial. In a meta-analysis based on 20,000 patients, pre-injury antiplatelets were associated with an increased risk of development of an intracranial hemorrhage after head trauma [38], particularly following clopidogrel [10, 16]. However, these studies mainly evaluated the relation between pre-injury antiplatelets in mild TBI in the emergency department setting with exclusion of patients who required emergency neurosurgery [10]. More recent studies have found no association between antiplatelets and hemorrhagic progression of post-traumatic lesions [7, 13, 28, 36], although Mathieu et al. found in a volumetric analysis that there was a small but significant increase in hemorrhage progression in cases with pre-injury antiplatelet treatment in the CENTER-TBI cohort, but it did not correlate with worse clinical outcome [22]. In the current study, we investigated patients with severe TBI admitted to the neurointensive care and found no significant association between antiplatelets and significant intracranial hemorrhage evolution. However, aspirin was the predominant antiplatelet, and only 5 patients received monotherapy with clopidogrel, why no further sub-analysis was done. It is possible that low-dose aspirin has a more limited impact on intracranial hemorrhage progression after head trauma, which could explain why antiplatelets did not correlate with worse intracranial hemorrhage evolution in our study.

However, previous studies have found clear evidence of intracranial hemorrhage progression following head trauma for patients who were pre-injury treated with vitamin K antagonists [6, 11, 28, 36]. Fabbri et al. did not find such an association [11], but they did not include those cases who required emergency neurosurgery, which could have excluded several cases with vitamin K antagonists. Consistent with the most previous studies, we found that pre-injury anticoagulant treatment was independently associated with an increased risk of significant hemorrhage evolution in severe TBI cases admitted to the NIC. Some recent studies have found a lower rate of severe intracranial hemorrhages with NOAC as compared to vitamin K antagonists [13, 28, 29]. However, only 6 patients were pre-injury treated with NOAC as monotherapy, why no further sub-analysis was performed.

Previous studies have reported an increased risk of severe intracranial hemorrhage progression in TBI for patients with a combination of antithrombotic agents [28]. In our study, the patients with a combination of antithrombotic agents had a trend towards increased hemorrhagic progression, but it did not reach statistical significance. This could be explained by the limited number of patients (*n* = 24) with such antithrombotic regimens and the heterogeneity in antithrombotic combinations in the current study.

In addition, we found that higher age was independently associated with significant hemorrhage evolution. Dunham et al. [7] found in a previous study including 198 TBI patients, in which most cases were mild to moderate, that pre-injury brain atrophy rather than antithrombotic agents predicted intracranial hemorrhage progression. It is likely that higher age with a corresponding brain atrophy may favor hemorrhage progression due to higher intracranial compliance with less hemorrhage tamponade. However, the lack of association between pre-injury antithrombotic agents and intracranial hemorrhage progression in their study could be explained by a smaller patient population.

### Antithrombotic agents and clinical outcome

The effect of pre-injury antithrombotic treatment on clinical outcome following TBI remains controversial [6, 7, 10, 13, 15, 18, 28, 36, 38]. Pre-injury antiplatelets are not associated with a higher mortality or worse clinical outcome in most studies [4, 14, 33, 36], although others have demonstrated an association with mortality [23]. However, the latter study did not adjust for confounding variables including age and comorbidities. Consistent with previous findings, we only found a significant association between antiplatelets and higher mortality and a decreased rate of favorable outcome in the univariate analysis, but not after adjustment for age and comorbidities. Active antiplatelet reversal was uncommon in our study, and less than 10% were treated with platelet transfusion/desmopressin, while antiplatelet withdrawal alone was the predominant management. Since pre-injury antiplatelet treatment was not associated with a worse intracranial hemorrhage evolution despite a low rate of reversal treatments, this supports that antiplatelet withdrawal is usually sufficient. However, interestingly, Barletta et al. found in a recent study that desmopressin treatment was significantly associated with lower intracranial hemorrhage progression after head trauma for patients with pre-injury antiplatelets, indicating a small benefit of desmopressin for these patients [2]. Future trials are needed to better determine when antiplatelet withdrawal is sufficient and if or when platelet transfusion and desmopressin are indicated. Altogether, these findings indicate that pre-injury antiplatelets are safe and do not generally have a significant impact on intracranial hemorrhage evolution and clinical outcome. However, the validity of these findings might be limited to low-dose aspirin, considering the low incidence of other antiplatelets.

Vitamin K antagonists are associated with increased mortality and unfavorable clinical outcome in many TBI studies [4, 11, 14, 28, 29, 36], but we and others have found no such associations [18, 22]. In the current study, anticoagulants were associated with a higher mortality and a decreased rate of favorable clinical outcome in the univariate analysis, but not after adjustment for age, comorbidities, and mechanism of injury. It has clearly been demonstrated that early reversal of vitamin K antagonists reduces hemorrhage progression and mortality [15] and it is possible that early reversal, in addition to the availability of emergency neurosurgery, may compensate for the increased risk of hemorrhagic progression. Some recent studies indicate that NOACs are associated with better clinical outcome than vitamin K antagonists after head trauma [28, 29]; however, due to the limited number of patients with pre-injury NOAC in the current study, we could not proceed with such a sub-analysis. Altogether, anticoagulants were independently associated with significant intracranial hemorrhage evolution, but not with worse clinical outcome. It is likely that early reversal of anticoagulants, emergency neurosurgery, and neurointensive care may compensate for the adverse effects of antithrombotic agents.

Furthermore, previous studies have found a worse clinical outcome for patients with a combination of antithrombotic agents [28]. This was not evident in the current study, but as outlined above, this could be explained by the heterogeneity of antithrombotic types and the limited number of patients with combined antithrombotic agents in the current study.

In addition, the role of tranexamic acid after TBI has gained interest, but the results from the CRASH-3 study are not in clear favor for early tranexamic acid treatment since only a sub-group of patients showed a modest decrease in mortality and other studies have followed where authors report no effect or increased mortality after tranexamic acid treatment in severe TBI [8, 27]. However, it is not ruled out that such treatment may be particularly important for a subgroup with pre-injury increased coagulopathy.

### Limitations

First, the patients included in the study were selected for admission to our tertiary neurointensive care unit if a favorable prognosis was considered possible. It is possible that some patients with antithrombotic agent-induced coagulopathy were excluded from neurointensive care admission and hence to the current study due to severe intracranial hemorrhages with a poor prognosis. The validity of our findings is therefore limited to those considered to benefit from neurointensive care. Second, although we included a significant number of patients (*n* = 844) over a wide time interval of 11 years, there was only a limited number of patients with clopidogrel and NOAC. This prohibited us from proceeding with further sub-analyses on their association with intracranial hemorrhage progression and clinical outcome. This also limits the validity of our findings to mostly aspirin and vitamin K antagonists. Third, there was great variation in the treatment of pre-injury antiplatelet treatment. It is possible that more aggressive reversal strategies were employed in cases with more severe intracranial hemorrhage evolution due to the concurrent coagulopathy or more severe traumatic forces to the head. Fourth, due to the large amount of radiological images evaluated in the current study, the analyses of hemorrhagic progression were only performed by one of the authors (TSW) as it was very time-consuming. The reliability of the radiological assessments is therefore limited to some extent. Fifth, our definition of intracranial hemorrhage evolution was based on a clinical point of view of significant hemorrhage progression, but it is possible that also antiplatelets would have been associated with a slight increase in clinically silent intracranial hemorrhage progressions. Sixth, groups 3 and 4 (emergency neurosurgery and early total brain infarction after the first CT, respectively) could in some cases represent progression of brain edema/injury rather than hemorrhage progression. We took this into account to some degree by evaluating if excluding those patients with presumed severe brain edema development (those operated with primary DC and those in group 4) from the regressions in Table 3 had any impact upon the results, but found no such effect.

## Conclusions

Antithrombotic agents are nowadays common, and almost one-fifth of the patients admitted for severe traumatic brain injury to our neurointensive care unit were pre-injury treated with such agents. In the majority of the patients with pre-injury antiplatelets, their medication was withdrawn but not treated with reversal agents, whereas almost all patients with vitamin K antagonists were treated with reversal agents. Those with pre-injury anticoagulants, but not those with pre-injury antiplatelets, were at higher risk for a more severe intracranial hemorrhage evolution. Patients with pre-injury antithrombotic agents had a higher risk for mortality and a decreased rate of favorable outcome compared to those without antithrombotic agents, but not in multiple regression analyses, as the former association was rather explained by higher age and more extensive comorbidities. This highlights that patients with severe traumatic brain injury who are pre-injury treated with antithrombotic agents, particularly anticoagulants, are at increased risk of severe intracranial hemorrhage evolution, but management, including anticoagulant reversal, emergency neurosurgery, and neurointensive care, may be important to achieve favorable outcome in these patients.

## Supplementary Information

ESM 1 (DOCX 16.8 kb)
