# Supplementary material for: Pre-injury antithrombotic agents predict intracranial hemorrhagic progression, but not worse clinical outcome in severe traumatic brain injury
Source: Acta Neurochir (Wien). 2021 Mar 26;163(5):1403–13. doi: 10.1007/s00701-021-04816-0 (PMC8053649; doi:10.1007/s00701-021-04816-0)
Supplement: Supplementary file 1 — (DOCX 16.8 kb) [file 701_2021_4816_MOESM1_ESM.docx]

**Appendix A. Antithrombotic agents in relation to mortality and favorable clinical outcome – a multiple logistic regression analysis including the IMPACT core variables**

| **Variables** | **Regression 1 – mortality** | | **Regression 2 – mortality** | |
| --- | --- | --- | --- | --- |
|  | OR (95%CI) | p-value | OR (95%CI) | p-value |
| Age | **1.05 (1.03-1.07)** | ***0.001*** | **1.05 (1.03-1.07)** | ***0.001*** |
| Charlson co-morbidity index | **1.55 (1.30-1.85)** | ***0.001*** | **1.57 (1.31-1.88)** | ***0.001*** |
| GCS M | **0.56 (0.47-0.66)** | ***0.001*** | **0.55 (0.47-0.66)** | ***0.001*** |
| Pupillary status (abnormal) | **2.87 (1.62-5.08)** | ***0.001*** | **2.84 (1.60-5.04)** | ***0.001*** |
| Antithrombotic agent (yes) | 1.11 (0.63-1.96) | 0.72 | NA | NA |
| Antiplatelet (yes) | NA | NA | 0.98 (0.47-2.06) | 0.96 |
| Anticoagulant (yes) | NA | NA | 1.18 (0.61-2.31) | 0.62 |
| Combination of antithrombotics (yes) | NA | NA | 0.76 (0.24-2.45) | 0.76 |
| **Variables** | **Regression 1 – favorable outcome** | | **Regression 2 – favorable outcome** | |
|  | OR (95%CI) | p-value | OR (95%CI) | p-value |
| Age | **0.96 (0.95-0.97)** | ***0.001*** | **0.96 (0.95-0.97)** | ***0.001*** |
| Charlson co-morbidity index | **0.80 (0.68-0.93)** | ***0.005*** | **0.77 (0.65-0.91)** | ***0.002*** |
| GCS M (1-6) | **1.93 (1.63-2.29)** | ***0.001*** | **1.94 (1.64-2.30)** | ***0.001*** |
| Pupillary status (abnormal) | **0.39 (0.23-0.65)** | ***0.001*** | **0.39 (0.23-0.66)** | ***0.001*** |
| Antithrombotic agent (yes) | 1.14 (0.69-1.87) | 0.61 | NA | NA |
| Antiplatelet (yes) | NA | NA | 1.31 (0.70-2.46) | 0.40 |
| Anticoagulant (yes) | NA | NA | 1.05 (0.56-1.96) | 0.89 |
| Combination of antithrombotics (yes) | NA | NA | 1.77 (0.59-5.31) | 0.31 |

The multiple logistic regression analyses describe the explanatory variables for mortality and favorable outcome, respectively. Pre-injury treatment with antithrombotic agents was grouped as one entity in regression 1, whereas different antithrombotic subtypes (antiplatelets, anticoagulants, and having a combination of antithrombotics) were analyzed in regression 2. CI = confidence interval. NA = not applicable.
